# Analysis of a marine phototrophic biofilm by confocal laser scanning microscopy using the new image quantification software PHLIP

**DOI:** 10.1186/1472-6785-6-1

**Published:** 2006-01-16

**Authors:** Lukas N Mueller, Jody FC de Brouwer, Jonas S Almeida, Lucas J Stal, João B Xavier

**Affiliations:** 1 Institute for Molecular Systems Biology, ETH Hönggerberg, CH-8093 Zürich, Switzerland; 2 Instituto de Tecnologia de Quimíca e Biológica, Universidade Nova de Lisboa, 2781-901 Oeiras, Portugal; 3 Scottish Association for Marine Science, Oban, Argyll, Scotland, PA37 1QA, UK; 4 Department of Biostatistics, Bioinformatics and Epidemiology, Medical University of South Carolina, Charleston SC 29425, USA; 5 Center for Estuarine and Marine Ecology, Netherlands Institute of Ecology (NIOO-KNAW), PO Box 140, 440 0 AC Yerseke, The Netherlands; 6 Department of Biotechnology, Delft University of Technology, 2628 BC Delft, The Netherlands

## Abstract

**Background:**

Confocal laser scanning microscopy (CLSM) is the method of choice to study interfacial biofilms and acquires time-resolved three-dimensional data of the biofilm structure. CLSM can be used in a multi-channel modus where the different channels map individual biofilm components. This communication presents a novel image quantification tool, PHLIP, for the quantitative analysis of large amounts of multichannel CLSM data in an automated way. PHLIP can be freely downloaded from

**Results:**

PHLIP is an open source public license Matlab toolbox that includes functions for CLSM imaging data handling and ten image analysis operations describing various aspects of biofilm morphology. The use of PHLIP is here demonstrated by a study of the development of a natural marine phototrophic biofilm. It is shown how the examination of the individual biofilm components using the multi-channel capability of PHLIP allowed the description of the dynamic spatial and temporal separation of diatoms, bacteria and organic and inorganic matter during the shift from a bacteria-dominated to a diatom-dominated phototrophic biofilm. Reflection images and weight measurements complementing the PHLIP analyses suggest that a large part of the biofilm mass consisted of inorganic mineral material.

**Conclusion:**

The presented case study reveals new insight into the temporal development of a phototrophic biofilm where multi-channel imaging allowed to parallel monitor the dynamics of the individual biofilm components over time. This application of PHLIP presents the power of biofilm image analysis by multi-channel CLSM software and demonstrates the importance of PHLIP for the scientific community as a flexible and extendable image analysis platform for automated image processing.

## Background

Interfacial biofilms constitute an important reservoir of microbial life in aquatic systems. The main focus of biofilm research has been on the examination of bacterial biofilms (reviewed in [1]), which cause widespread problems in industrial fluid processing applications (reviewed in [2]) and play a major role in human infection diseases [3]. In general, growth of biofilms is initiated by the attachment of cells to a conditioned substratum, after which *in situ *growth of micro-organisms and production of extracellular matrix components build up the biofilm structure [4]. Many studies have investigated the physiology and structure of bacterial biofilms in order to understand the underlying processes of attachment, detachment and growth (reviewed in [5]).

In contrast to medical and industrial settings where biofilms generally develop in the dark, those present in freshwater and marine aquatic ecosystems are often exposed to sunlight. In these environments, biofilms are diverse species communities typically dominated by micro-algal consortia [6-9]. Phototrophic biofilms in aquatic environments represent an important carbon source for other trophic levels [10,11] and affect mass transfer processes at the ecosystem scale [8].

Confocal laser scanning microscopy (CLSM) is an important method for the study of biofilm structure. Since its first application [12], CLSM has become widely used to improve the understanding of the biofilm architecture [13]. CLSM is a non-destructive and non-invasive method with the capability to provide time-resolved three-dimensional images of biofilms. In addition, multiple fluorescent channels can be recorded simultaneously, which offers the possibility to directly observe the development of individual biofilm components [14]. Multi-channel observations of phototrophic biofilms take advantage of autofluorescence of the micro-algae or record the fluorescence signal of biofilm components labeled with specific markers. Analysis of CLSM images has shown that biofilm communities form highly structured microbial assemblies [15,16]. Studies using CLSM have further confirmed that the development of biofilms depends on various factors including mass transport properties [17] and have shown the importance of metabolic interactions within the microbial communities themselves [18].

In many studies, the analysis of CLSM data has been of rather qualitative than quantitative nature and consisted entirely of a visual image inspection [19,20]. This approach is however subjective and not feasible when large quantities of data have to be analyzed, which is often necessary to ensure the significance of the outcome of the analyses [21]. For quantitative analysis of CLSM data, computer software with different functionalities ranging from cell number counting [22] to the classification of bacteria morphotypes [23] are currently available and are increasingly being used. In order to address the necessity of a more directed morphological quantification of biofilm data, image analysis programs such as COMSTAT [24] and ISA [25] were developed to quantitatively analyze single channel 3D CLSM data of biofilm imaging by determining a set of morphological parameters. Despite the wide use of the available image analysis software in morphological investigations of bacterial biofilms [15,21,26,27], their application is restricted to the analysis of single-channel CLSM data at a time. This single-channel limitation falls short of the capabilities of modern CLSM devices and is a significant disadvantage since multi-channel images have to be quantified in separate analysis sessions and multiple channel analysis distinguishing various biofilm components cannot be approached comprehensively.

This communication describes a new image quantification package, PHLIP (PHobia Laser scanning microscopy Imaging Processor), which enables analysis of multi-channel CLSM data. PHLIP was developed in the scope of the EU/FP5 funded project PHOBIA (Phototrophic Biofilms and Its potential Applications [28]) and extends previous work on automated CLSM image analysis [29,30] by including a new set of tools to automatically quantify CLSM imaging in biofilm systems, necessary to produce statistically meaningful results. PHLIP is available as an open source project (see Materials and Methods) and encourages users to extend the program's capabilities. The potential of PHLIP is illustrated here in a study of the temporal development of a natural phototrophic biofilm.

### Implementation

The image analysis software PHLIP was implemented as a MATLAB package running under MATLAB (Release 13, The Mathworks™) on Windows, Linux and OSX platform and does not require additional toolboxes. PHLIP is released under an open source license and current software versions as well as the documentation and example image datasets can be downloaded from the project webpage [31]. The architecture of the program was developed with flexibility and extensibility in mind and its functionality can be easily expanded with new features. PHLIP therefore represents a platform for the integration of novel image processing operations without the need to code for import, export or preprocessing functions. Supplementary information regarding the program structure and guidance for the implementation of new image operations are available on the project homepage. Fig. 6 describes the data analysis flow of PHLIP and illustrates the individual image processing functions of the 5 distinct modules constituting the program.

### PHLIP-ML, a new standard for CLSM imaging data

Due to the lack of uniformity in image formats between the different CLSM vendors or even between different models from the same vendor, a new CLSM standard formatted as XML (eXtensible Markup Language) was developed. XML was chosen as its general acceptance as a standard to describe data in a vast number of fields [32], including generous support for development of converter from other formats. Basic image acquisition parameters (image resolution, scanning step etc.) and CLSM imaging data information (file names, number of channels etc.) are stored in the PHLIP-ML files. Optionally, PHLIP image analysis results can also be incorporated into PHLIP-ML. Detailed information about the PHLIP-ML data structure as well as its Document Type Definition file (dtd), graphical representations and example data sets can be retrieved from the PHLIP-ML webpage [33]. A universal converter *data2xml *is available on the PHLIP project homepage [34] and offers automated conversion of PHLIP supported CLSM formats into PHLIP-ML. For not supported CLSM formats, *data2xml *can be used to generate in an interactive process PHLIP-ML files from any kind of microscopic images without any previous XML knowledge.

### Import and pre-processing of CLSM data

In addition to the PHLIP-ML format, PHLIP currently offers direct support for the two CLSM models of Leica™ (Heidelberg, Germany), Leica TCS-NT and Leica TCS4D. The latter format is also used by the COMSTAT program [24], rendering PHLIP compatible to COMSTAT. In addition, the functionality of PHLIP can be easily extended to allow direct import of data from other CLSM formats. In the present version, PHLIP enables quantification of CLSM data sets containing up to four fluorescent channels.

The image pre-processing module offers several possibilities to pre-process the batch imaging data. A batch editing menu allows the user to select or remove image stacks from the data set. To save computer memory and reduce computational requirements, the cross section resolution of CLSM image stacks can be adjusted by skipping image cross sections within image stacks (Fig 6A). Furthermore, the microscope scanning direction can be defined which is important for the correct 3D reconstruction of image stacks from image cross sections files (Fig 6B). Additional pre-processing functions include adjustment of the biofilm carrier horizontal position within the 3D image stack (Fig 6C), which allows to define the deepest cross section containing any biofilm material, and pre-reading of the input files to ensure data availability.

### Thresholding and graphical user interface

Image segmentation is the process where every pixel with a grey value above a given threshold is classified as foreground and a pixel with value below a given threshold as background, resulting in a binary image. Selection of a threshold level is therefore an important step in the quantitative analysis of CLSM biofilm imaging as altering the threshold value will change the volume and morphology assigned to a given biofilm component [29]. Threshold determination is typically carried out by either manual assessment (e.g. [25,35,36]) or automated methods [29,37,38]. To our knowledge, there is no generally accepted method, manual or automated, that works accurately for every possible set of imaging data. For a discussion on the applicability of automatic thresholding see a study by Yang *et al *[37] in which several automated threshold selection methods are evaluated.

Automated thresholding algorithms have several advantages over manual selection methods but in our view there is no automated procedure that guaranties to work correctly with every image set. This is partially because of the wide variability in characteristics of images from different samples, e.g. in terms of image histograms or spatial distribution of features within the samples. Advantages of automated thresholding methods include the possibility of automating the full image processing procedure [30]. PHILP has been equipped with this automating capability allowing the processing of large image datasets without user intervention and eliminating subjectivity associated with visual threshold selection by an operator [29,38]. However, the use of automated threshold methods should always be complemented with some degree of supervision, which reinforces the critical value of multi-channel visualization methods to inspect the computed thresholds.

PHLIP offers users the choice of both manual and automated threshold selection. For manual threshold selection, PHLIP provides a multi functional graphical user interface (GUI) to assist in the visual determination of the threshold level to be used for every color channel of each dataset. For automated threshold selection, PHLIP implements a 3D extension of the Otsu algorithm [29]. Otsu is a well-established method that consists of a simple threshold selection procedure and, therefore, does not constitute a significant computationally burden to the image processing as a whole. This renders the method particularly suitable to image analysis systems, which will most likely be installed on personal computers. The method is based on discriminate analysis, which determines the variable that separates best between two naturally occurring groups. In the application of image analysis, a grey level value is selected, which separates the two groups of foreground and background grey level values. In PHLIP, the 3D Otsu threshold selection is applied to the CLSM data set to calculate thresholds independently for each color channel in each image stack.

With the threshold level being selected either manually or automatically, the GUI allows the user to view the different channels of an image stack and to browse through their cross sections, assessing the adequacy of the determined thresholds. Three different views of the image stack cross sections help to set, alter or check a threshold; an original grey level view, a view of the thresholded image and a combined view where the thresholded image overlays the original grey level image.

### Morphological characterization

Following binarization of the image through thresholding, biofilm parameters are calculated from the binary image stacks with a prior optional connected volume filtration (CVF) [24,30]. The CVF method removes "floating" foreground pixels, ensuring that every foreground pixel is connected to the substratum through the connection to other neighboring pixels. Application of the CVF operation is optional, as users may prefer to include relevant floating material in their quantitative analysis depending on the characteristics of the system being analyzed. Presently, PHLIP can quantify 10 different biofilm features, which are classified into 6 single-channel (A – F), 2 two-channel (G, H) and 5 all-channel measurements.

*A. Biovolume: *the biovolume, V, is the number of foreground pixels, N, in an image stack multiplied by the voxel volume, which is defined as the product of the squared pixel size, px, and the scanning step size, zStep [35].

*B. Substratum coverage: *PHLIP calculates the fraction of pixels occupied by biofilm material for each image cross section [30]. The fraction, F(z), is defined as the ratio of foreground pixels to the total number of pixels for a given cross section and is then transformed to percentage, C(z).

*C. Area to volume ratio: *the surface area, A, of an image stack is the number of foreground pixels which are connected to at least one neighboring background pixel. The final value is then obtained by calculating the ratio A to V [24].

*D. Spatial spreading: *this function characterizes the spreading of biovolume in space. Three different values are calculated; horizontal spreading (in xy direction), S_xy_, vertical spreading (in z direction), S_z_, and total spreading (in xyz space), S_xyz_. Initially, three distributions D_x_, D_y _and D_z _containing the values of the x, y or z coordinates of each foreground pixel in a given image stack are created. The variances σ_x_, σ_y _and σ_z _are then calculated from D_x_, D_y _and D_z_. S_z _results directly from σ_z _(1) while equation 2 combines σ_x _and σ_y _to obtain S_xy_. S_xyz _is determined by σ_x_, σ_y _and σ_z _(3). All three parameters are dimensionless:

S_z _= σ_z _    (1)

S_xy _= σ_x_^2 ^+ σ_y_^2 ^    (2)

S_xyz _= σ_x_^2 ^+ σ_y_^2 ^+ σ_z_^2 ^    (3)

*E. Mean thickness and roughness: *these parameters are widely used to describe the morphology of the biofilms. The function first applies a height projection transformation to the image stack where for every point in the xy plane the maximal height h of the corresponding foreground pixels in z direction is stored. The average of the resulting distribution of pixel height h is then calculated and represents the mean thickness, M, [30]. To obtain the roughness coefficient, R, the standard deviation of the distribution is divided by M [24].

*F. Fractal dimension in 2D: *the fractal dimension parameter calculates a value that varies between 1 and 2 and describes the roughness of the biofilm boundary between foreground and background pixel in a cross-section at height z. Higher values of the fractal dimension parameter indicate a rougher biofilm boundary. The fractal dimension parameter is calculated as described by Yang *et al *[25].

*G. Co-localization in 3D: *the co-localization in 3D operation calculates the percentage of overlapping biovolume in two selected channels. The co-localization volume, V_col_, is calculated by counting the presence of foreground pixels located at identical xyz positions in two channels. This is divided by the total biovolume of both channels [39].

*H. Co-localization in 2D: *the co-localization can also be studied in 2D for each cross section located at height z. Analogous to operation F, the fraction of co-localized to the total occupied area in 2D of two channels is calculated for every cross section [39].

*All channels operations: *biovolume, substratum coverage, area to volume ratio, spatial spreading, mean thickness and roughness analyses in the all channel mode are calculated by the same mathematics as their single channel operation analogues A to E. These operations work with a new 3D stack containing all biofilm elements from each channel where overlapping elements between the different channels are removed to avoid double counting.

### Data output

PHLIP offers the possibility to save the calculated results in HTML, text and PHLIP-ML format. The HTML output is a formatted file presenting the results in tables and also contains plots for operations B, F and H. This data file can be opened by most spreadsheet programs and facilitates further analysis of the obtained results. Alternatively to the HTML format, analysis results can be formatted in a tab-delimitated matrix into a simple text file. As described above, the calculated results can also be saved in XML format using the PHLIP-ML data structure. This feature has the advantage that defined thresholds from an earlier analysis are stored in the PHLIP-ML file and can be re-loaded into PHLIP to continue a previous analysis procedure. In addition, PHLIP-ML formatted CLSM analysis results represent a compact way to archive imaging data or to transfer calculated results to other downstream data analysis applications.

### Experimental set-up for the growth of phototropic biofilms

Natural phototrophic biofilms were grown in 70 1 containers that were fed by a flow of fresh unfiltered natural seawater (Oosterschelde, the Netherlands) at a dilution rate of 0.43 h^-1^. Microscope glass slides were cleaned using 70% ethanol, dried and subsequently placed vertically in the water. The system was exposed to a 14:10 h light:dark regime at an incident photon irradiance of 40 *μ*mol·m^-2^·s^-1^. Pumps circulating water at a rate of 300 1· h^-1 ^ensured continuous turbulent mixing. Sampling was performed over a period of 44 days. On 7 sampling days (T = 3, 5, 10, 18, 25, 31, 44 days), 3 glass slides were randomly removed from the water reservoir 4 h after the onset of light and prepared for CLSM analysis.

Glass slides were rinsed twice with 1 ml of PBS (phosphate buffered saline: 0.4 g·l^-1 ^KC1, 12.6 g·l^-1 ^NaC1, 1.6 g·l^-1 ^Na_2_HPO_4 _·2H_2_O, 0.25 g·l^-1 ^KH_2_PO_4_, pH = 8). Excess fluid was removed by using Whatman GF/B filter paper (Maidstone, UK). Subsequently, the slides were incubated in the dark for 20 min using 200 *μ*l of the lectin concanavaline A (ConA, Molecular Probes, Eugene, Oregon, USA) at a concentration of 100 *μ*g·ml^-1 ^(in PBS). Excess ConA was removed by two rinses of 1 ml PBS and one rinse with 1 ml TRIS (18.6 g·l^-1 ^EDTA, 0.158 g·l^-1 ^TRIS, 20 g·l^-1 ^NaC1, 0.4 g·l^-1 ^KC1). Thereafter, slides were incubated for 2 min using 200 *μ*l, 5 mM syto64 (Molecular Probes) dissolved in TRIS-buffer to stain bacteria. Excess syto64 was removed by 2 rinses of 1 ml TRS and one rinse of 1 ml PBS.

CLSM was performed using a TCS-NT microscope (Leica, Heidelberg, Germany) equipped with an Argon-Krypton laser. Biofilms were examined using a HCX APO L 40 × 0.8 water-immersion lens. Table 2 summarizes excitation wavelengths and emission filters of the CLSM. Seven random spots were measured at each of the 3 replicate glass slide, resulting in 21 replicate measurements per sampling day. In addition, reflection images were measured on selected biofilm samples. For this purpose, an RT 30/70 filter was used and the image stacks were recorded under identical conditions (i.e. similar area and vertical resolution) compared to the 3 fluorescence channel analysis.

### Statistical analyses

One and two way nested analysis of variance (ANOVA) designs were used to test the effects of days and sampling slides on the biofilm parameters obtained from the PHLIP analysis. For one way ANOVA, slides were nested within days. For two way ANOVA, slides were nested within the interaction between days and channels. Analyses were performed on box-cox transformed data [40]. In addition, regression analysis was performed on the all-channel parameters. Either linear or exponential decay functions were used to fit the datasets. Analyses of variance were performed using Statistica 6.1, while regression analysis was performed using the software package Origin 6.0.

## Results

The functionality of PHLIP is illustrated here by analyzing stacks of images acquired by CSLM at different maturation stages of a phototrophic biofilm. The quantitative description of biofilm morphogenesis with regard to the distribution of three dominant components, bacteria, micro-algae and extracellular polymeric substances (EPS), was pursued with the tandem goal of describing the biological process and establishing a multi-channel image analysis methodology.

**Figure 1:**
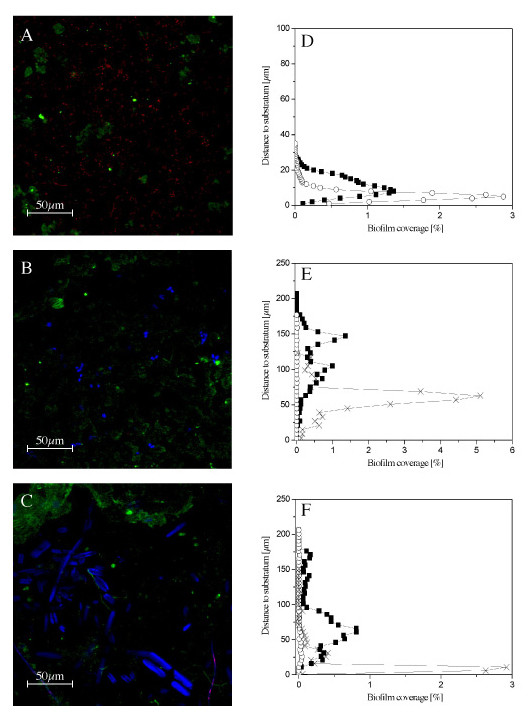
Maximum intensity images (A, B, C) and vertical profiles of biofilm coverage analyzed by PHLIP (D, E, F) of a biofilm grown on glass slides in natural seawater (Oosterschelde, the Netherlands). Images and vertical profiles are shown for representative samples measured at days 3 (A, D), day 25 (B, E) and day 44 (C, F). The red channel and open circles represent bacteria stained with syto64, the green channel and black squares represent EPS labeled with ConA and the blue channel and crosses show chlorophyll *a *autofluorescence. CLSM maximum intensity images represent areas of 250·m × 250·m.

A selection of maximum intensity projection images and the accompanying vertical profiles from image stacks recorded at different time points during the experiment are shown in Fig. 1. After a period of 3 days (Fig. 1A), biofilms were dominated by bacteria that were partly embedded in ConA-labeled EPS of the biofilm matrix. During the course of the experiment the contribution of bacteria decreased while the biomass of micro-algae increased. Changes in the morphology of the chloroplasts indicated a succession in the algal community with a general shift towards larger species (Fig. 1B,C). The presence of EPS was observed throughout the time series. EPS structures were visible mainly as bright spots or amorphous organic material. Occasionally, cell surface material of algal cell was stained with ConA. The thickness of the biofilms increased from about 30·m after 3 days of incubation (Fig. 1D) to 200·m in 44-day-old biofilms (Fig. 1F). Bacteria were only observed as a significant fraction in the 3-day-old biofilm where they were present near the substratum (Fig. 1D). The vertical distribution of micro-algae was variable. Part of the algal community was present close to the substratum while the remaining part extended into the biofilm (Fig. 1E,F). Vertical profiles showed considerable variation between different measurements, but EPS generally formed the thickest and outermost layer of the biofilm. A maximum of approximately 5% of the available surface was occupied by biofilm components after 44 days of incubation.

**Figure 2:**
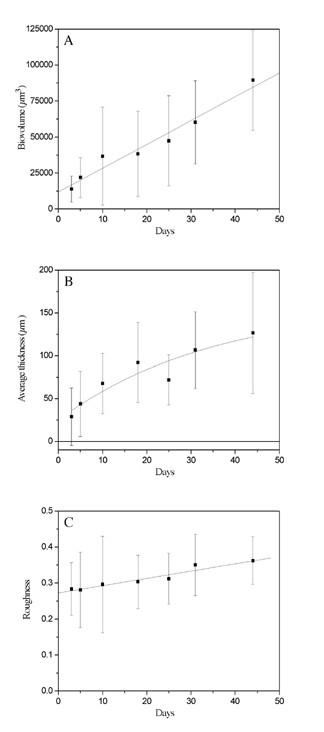
Temporal development of a natural phototrophic biofilm grown on glass slides in natural seawater (Oosterschelde, the Netherlands). All-channel image analyses of (A) biovolume, (B) average thickness and (C) roughness. Values indicate mean ± S.D., (n = 21).

In a more comprehensive approach, the development of the biofilm was analyzed by CLSM measuring 7 replicate points on each of 3 replicate glass slides. Image analysis results (Table 1) were in good agreement with qualitative observations. The single channel parameters showed the temporal development in chlorophyll *a *and syto64 signal representing growth of the phototrophic community and the decrease of bacteria, respectively. Growth of the micro-algae dominated the development of biofilm. This led to a significant increase in biovolume, thickness and roughness of the biofilm (Fig. 2A,B,C). Biomass-related and morphological parameters of ConA-stained organic matter did not vary over time while those related to the vertical distribution (i.e. average thickness and vertical spreading) increased significantly. Two channel operations indicated that co-localization between channels was generally low with up to 5.2 % co-localization occurring in the three dimensional image stacks. A general trend in the results of the co-localization analysis showed that co-localization of channels ConA and syto64 was always higher then in channels ConA and chlorophyll *a*. This identifies a preferential spatial association of EPS with bacteria in relation to association of EPS with diatom, suggesting a bacterial origin of EPS.

The quantitative description of biofilm morphology performed by PHLIP analysis enables the testing for changes of distinct quantifiers in response to different condition. Equally, PHLIP allows to characterize the variability of these quantifiers within or between distinct biofilm communities. Statistical evaluation of the dataset by nested ANOVA, which was used here to illustrate the functionality of PHLIP, revealed significant differences between the slides as well as an effect of time. The results of these experiments will demonstrate the insights of the quantitative parameters extracted from CLSM image analysis. Temporal variability was observed for the all-channel operations as well as for the separate CLSM channels. This suggested that the placement of the slides in the experimental setup was a factor that determined the development of the biofilms, probably cause by spatial variations in light and/or flow conditions. However, by using the nested ANOVA-design it was possible to discriminate between different sources of variation. Hence, statistically meaningful results were obtained with respect to the variables time and channels. All-channel analyses resulted in a significant linear increase in biovolume (Fig. 2A, R^2 ^= 0.9704, p < 0.001). Biofilm thickness (Fig. 2B) also increased over time (p < 0.01) but the temporal evolution was best predicted by an exponential decay function (R^2 ^= 0.8855, y = 0.33–0.29xe^(-x/16647)^). Even though a linear correlation between roughness and days of incubation (Fig. 2C, R^2 ^= 0.9328) was observed, statistical analysis showed that this trend was not significant (p = 0.57).

**Figure 3:**
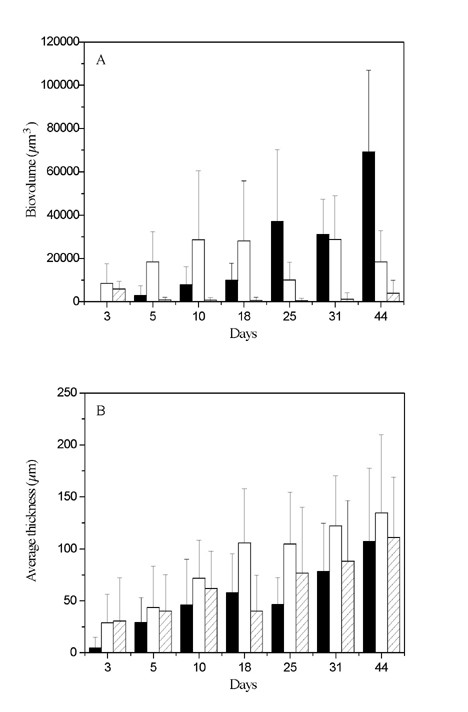
Temporal development of a natural phototrophic biofilms grown on glass slides in natural seawater (Oosterschelde, the Netherlands). Image analyses of separate channels representing chlorophyll *a *fluorescence (black bars), EPS stained with ConA (open bars) and bacteria stained with syto64 (dashed bars). (A) biovolume and (B) average thickness. Values indicate mean ± S.D., (n = 21).

**Figure 4:**
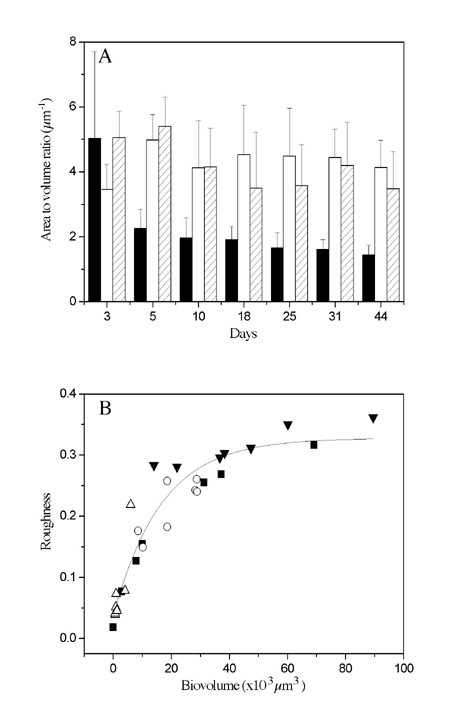
Development of morphological parameters analyzed from a phototrophic biofilm grown on glass in natural seawater (Oosterschelde, the Netherlands). (A) Temporal development of area-to-volume ratio. Separate channels represent chlorophyll *a *fluorescence (black bars), EPS stained with Con A (open bars) and bacteria stained with syto64 (dashed bars). Values indicate mean ± S.D, (n = 21). (B) The correlation between biofilm roughness and biovolume. Filled squares: chlorophyll *a *fluorescence, open circles: ConA-fluorescence, open triangles: syto64, filled triangles, all channel signal.

Analysis of the information from different channels acquired by CLSM revealed that different biological components in the biofilms (i.e. bacteria, micro-algae and the ConA-stained EPS) followed different dynamics in time (Fig. 3, 4). Biovolume of the EPS was approximately constant over time (p = 0.45), while the bacterial signal decreased (p < 0.001). In contrast, a steady increase (p < 0.001) was observed for the chlorophyll *a *biovolume (Fig. 3A). Average thickness of the biofilm, as determined from each individual component, increased over time for all channels (p < 0.001 for chlorophyll *a *and ConA, p < 0.05 for syto64), but thickness of ConA was significantly higher compared to the chlorophyll *a *and syto64 signals (p < 0.001 and p < 0.05, Fig. 3B). This indicated that some EPS always exists in the region of the biofilm above the other components monitored, i.e. micro-algae and bacteria.

Variations in the morphological parameters area-to-volume ratio and biofilm roughness occurred over time (Fig. 4). Area-to-volume ratios of chlorophyll *a *showed a steady decrease after 5 days of incubation (p < 0.001, Fig. 4A). This is in agreement with the changes in morphology of the chloroplasts as observed in the CLSM images (Fig. 1), indicating a succession of diatoms over time from small species to larger species. In addition, area-to-volume ratios of the bacterial signal varied over time (p < 0.05) although no clear temporal trend was found. Finally, the EPS signal was constant and showed a rather diffuse distribution of signal compared to the chlorophyll *a *autofluorescence. Biofilm roughness of the separate biofilm components gave comparable patterns to the temporal changes in biovolume. When the estimates of biofilm roughness were plotted as a function of biovolume, a correlation was found between the parameters (Fig. 4B). The exponential decay function that was fitted through the data explained 91% of the variation.

**Figure 5:**
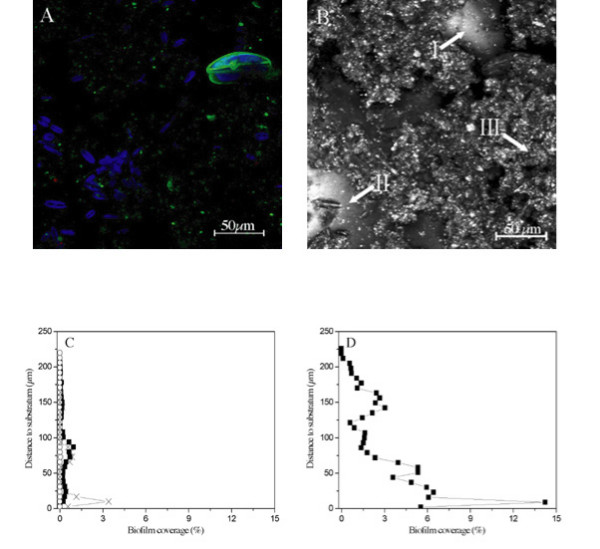
Maximum projection images of a biofilm grown on glass slides after incubation in natural seawater (Oosterschelde, the Netherlands). Visualization of bacteria, EPS and chlorophyll *a *(A) and reflection image (B) of a 31 day old biofilm. The red channel represents bacteria stained with syto64, the green channel represents EPS labeled with ConA and the blue channel shows chlorophyll *a *autofluorescence. In addition, the depth profiles of biofilm coverage for ConA (filled squares), syto64 (open circles) and chlorophyll *a *(crosses) are given (C) as well as the depth profile for the reflection signal (D). The CLSM images represent areas of 250·m × 250·m. White arrows indicate the different reflective structures containing bare glass substratum (I), silica frustules of diatoms (II) and amorphous material (III).

**Figure 6:**
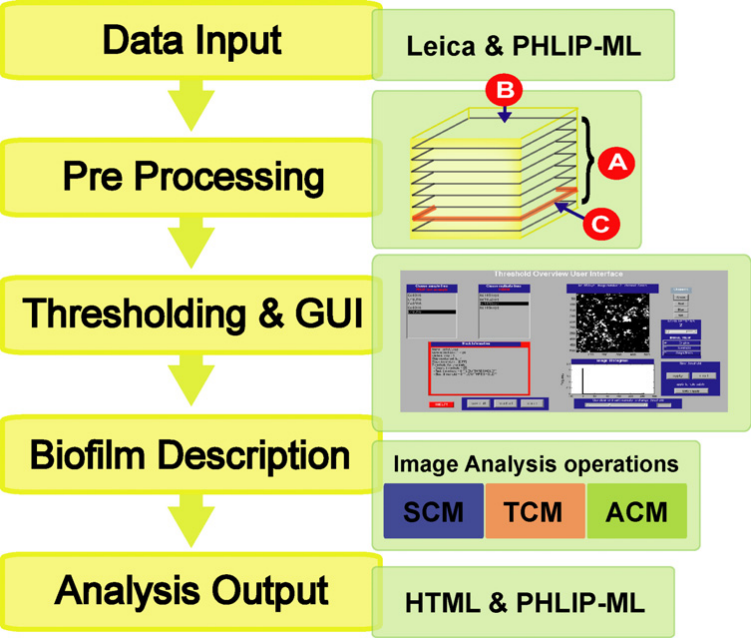
Data processing flow of PHLIP. The program flow is defined by the sequence of calls to the individual functions (green background) of the available image processing modules (yellow). Each module represents a different image processing operation type. The illustrations symbolize optional choices from a set of functions by the user, arrows represent strict program routines. GUI: graphical user interface, SCM: single-channel, TCM: two-channel, ACM: all-channel measurements.

In addition to the quantification of biological components, which included bacteria, micro-algae and EPS, reflection images of biofilms visualize solid inorganic material (Fig. 5) and can be similarly analyzed using PHLIP as illustrated here. The quantified signal reflects the surface of the three dimensional objects from the inorganic material. Different reflective materials were distinguished including the bare glass substratum (I), silica frustules of diatoms present in the biofilm (II) and considerable amounts of amorphous material (III). A comparison of vertical profiles of the organic and inorganic parts of the biofilm indicated that the reflection signal (indicative for inorganic material) was consistently higher than the organic matter throughout the depth of biofilm. Biovolume estimates showed that for the image stack in Fig. 5, about 85% of the biofilm constituted of inorganic reflective material, while organic material represented the remaining 15% of the total biovolume. This percentage should be regarded as a rough estimate because inorganic particles represent opaque surfaces that are not penetrated by the laser. In addition, the glass surface gives a strong reflectance signal that is not a part of the biofilm itself. The value for the inorganic matter contribution of 85 % agrees with determinations of dry weight and ash free dry weight contents of the biofilm (data not shown). These measurements indicated that the contribution of inorganic material to the biofilm was 58% after 3 day of incubation and increased to an average value of 78 ± 4% during the course of the experiment. Hence, both confocal as well as dry mass determinations indicate a significant contribution of inorganic material in this phototrophic biofilm.

## Discussion

This section separately covers the results describing the biofilm development followed by a discussion of the quantitative methodology implemented in PHLIP.

### Biofilm development

The temporal development of a natural phototrophic biofilm was characterized by a linear increase in biovolume. Biofilm thickness increase leveled slightly off with time, which is explained by the fact that the laser of the CLSM did not penetrate anymore all the way down to the substratum of the biofilm. Eventually, phototrophic micro-organisms (dominated by diatoms) became the dominant component of this biofilm. A succession of the phototrophic community was evident from small to larger species, both qualitatively from visual inspection of images (Fig. 1) and quantitatively from image analysis using PHLIP (Fig. 3A). A possible explanation for this observation is that smaller algae are more opportunistic species adapting more easily, while the bigger ones need a conditioned environment to thrive. During biofilm development, the different biological components of the biofilms depicted partial stratification (Fig. 1D,E,F). Bacteria were only observed in significant quantities at day 3 and were located close to the substratum. Micro-algae generally inhabited the surface to intermediate layers of the biofilm. The outermost layer of the biofilm almost exclusively consisted of EPS, which protruded into the surrounding water (see also Fig. 3B). Micro-stratification has been observed previously in river stream biofilms in which the layer of bacterial was close to the substratum and separated from canopy of micro-algae by an EPS-dominated intermediate layer [8]. These results confirm that EPS may be heterogeneously distributed in biofilms. EPS production is considered an important way for biofilm inhabiting organisms to structure their environment [41]. Although EPS was observed as an integral part of the biofilm, this study did not clearly identify the sources of this extracellular material. As mentioned above, two-channel co-localization results from PHLIP analysis (Table 1) show higher values for EPS-bacteria co-localization (ConA-syto64) than for diatom-EPS co-localization (ConA-chla). This slight preferential spatial association of bacteria and EPS suggests that the EPS in this biofilm may be in part of bacterial origin. Visual inspection of images, however, does not provide confirmation that EPS is associated with micro-organisms such as bacteria (Fig. 1A) or diatoms (Fig. 5A). It cannot be excluded that the exopolymeric material that was detected by concanavalin A was not produced by micro-organisms in the biofilm but derived from material attached to particles that were subsequently incorporated into the biofilms (Fig. 5). It is also possible that the organisms had moved deeper into the biofilm leaving the secreted EPS behind. In either case, the stratification of biofilm components and the presence of EPS at the biofilm-water interface are likely to influence mass transfer processes within the biofilm systems [41].

The all-channel analyses showed gradual temporal increases in biovolume and average thickness (Fig. 2). Although this could suggest that development of this biofilm occurred with a rather constant level of heterogeneity, single-channel analyses indicated otherwise. Within the biofilms, bacteria, micro-algae and EPS components followed different dynamics in time (Fig. 3, 4). The appearance of a phototrophic community was preceded by colonization of the glass surface by bacteria which is in agreement with Chan *et al *[42] who found that bacteria are generally the first colonizers in the development of marine biofilms. During the experiment a clear change was observed from a heterotrophic system dominated by bacteria to an autotrophic system dominated by diatoms. A similar type of dynamics was found for the development of phototrophic freshwater biofilms grown in drinking water basins [9]. Algal and bacterial biomasses were not coupled and bacterial biovolume remained invariably low while micro-algal biomass increased rapidly (Fig. 3A). This is in disagreement with several studies in pelagic systems [43], river stream biofilms [8,44] and marine biofilms [45] where a coupling between algal and bacterial biomass was observed. However, other studies dealing with development of phototrophic biofilms in estuarine systems supported the observation that during phototrophic biofilm development, bacterial biomass remains low [6,42]. In marine phytoplankton aggregates, uncoupling of bacteria and algae has been found as a result of grazing on bacteria [46] or insufficient bioavailable algal derived dissolved organic matter [47]. In the present study, the effect of grazing was not specifically investigated. However, microscopic observations showed the presence of organisms in the biofilms that may have utilized biofilm components as a food source. In addition, CLSM analysis of lotic biofilms indicated that grazing greatly impacted on the contribution of biological components and on the morphology of biofilms [11]. Although the structure of the biofilm was generally highly porous, syto64 may not have been penetrated well in the compact parts of the biofilm [9]. Therefore, we could not exclude the possibility that bacterial biovolume was underestimated. This would mean that bacteria were mainly present in EPS aggregates where label penetration is hampered. Other studies suggest that bacteria in natural biofilms are mainly associated with the substratum [48] or distributed in a shell-like manner in outermost regions of biofilms [16]. In addition, previous studies have shown that the contribution of bacteria to phototrophic biofilms was low and typically varied between 0.01 and 5% [16,48,49]. The proportion of bacteria found in this study was in the same range after the phototrophic biofilm was fully developed (1.0–4.9%). This suggests that the contribution of bacteria in phototrophic biofilms may be low when compared to the algal and EPS components, which represented 20–76% and 19–77% of the biofilms, respectively.

The various temporal patterns of individual biofilm components during development of biofilms were also reflected in the morphological characterization.

Roughness coefficients for the bacterial and algal components varied to a large extend whereas those of the EPS matrix were repeatedly high. This supports the view that EPS is an important component defining the structural characteristics of this type of biofilm [41,50]. In spite of the different temporal dynamics, a consistent correlation was found between biovolume and roughness of the various biofilm components showing that the biofilm system studied here increased in heterogeneity during its development. However, this does not seem to be a general feature of (phototrophic) biofilms. For example, in phototrophic river stream biofilms roughness coefficients decreased during biofilm development [8]. The mechanisms that determine the morphology of biofilms are currently not understood. It has been observed that morphological development of biofilms is species dependent [24]. Moreover, modeling as well as empirical studies have indicated that biofilm morphology is influenced by externally imposed constraints including hydrodynamic forces and substrate availability [51-53].

Besides the presence of biological components, the biofilm under study contained considerably amounts of mineral particles as reported for river snow aggregates [19] and river biofilms [8]. It was estimated that as much as 85% of the biofilm mass was made up by inorganic material, which was either biogenic (e.g. silica, carbonate) or non-biogenic (e.g. silts) in nature. Using chlorophyll *a *data of the biofilm (not shown) and a Si : chla ratio of 10.9 [54], it was estimated that silica represented approximately 1% of the mineral mass present in the mature biofilm. This was derived from the phototrophic community that was dominated by diatoms (based on pigment analysis; data not shown). Hence, only a minor part of the inorganic material present in the biofilm was derived from the living phototrophic biomass. Microscope observations in the reflecting mode confirmed this conclusion by showing a predominance of amorphous structures. These structures were visible in the biofilms (Fig. 5B) and may have served to increase the surface area allowing more efficient attachment of micro-organisms to the substratum. In addition, the incorporation of silt particles in biofilm may provide an additional source of nutrients. This was observed in sedimentary diatom biofilms were a positive feedback was found between diatom growth and silt accumulation [55]. Indeed, increased nutrient concentrations have been reported in estuarine fouling biofilms compared to the ambient water [6].

### CLSM quantification with PHLIP

As shown in previous applications of PHLIP [56,57], we demonstrated here the usefulness of the presented program by the analysis of a large amount of multi-channel CLSM data. In contrast to the recently published program ISA-3D [25], PHLIP is free of charge and the user-friendly program design does not require any training course to understand the program handling. Table 3 in the supplementary material presents a feature overview of the image analysis software mentioned in this article. The recently formed IWA specialist group on biofilm structure aims to evaluate existing image analysis programs (COMSTAT, ISA-3D, PHLIP etc.) and it will be interesting to see the comparison of their performance. We hope that the IWA specialist group will recognize the usefulness of a common CLSM format and help to establish the PHLIP-ML standard within the biofilm community. Although PHLIP constitutes a fully functional image analysis software, the open source project PHLIP intends to further expand the program's capabilities. Current unstable versions offer support for CLSM data from cryo sections including automated stack tilting and incorporate an extension of the fractal dimension parameter into 3D.

To meet the expectations of a novel image analysis software, PHLIP unifies a combination of concepts for automated quantification procedures [29,30,35] and its features address essential issues of morphological quantification for CLSM data of biofilms:

#### Formalism

Qualitative morphological analysis of biofilm is often based on subjective concepts. Image processing methods use a quantitative approach to formally and mathematically describe morphological traits [25].

#### Automation

PHLIP implements a fully automated image processing procedure, which removes any subjectivity inherited from operator intervention. These batch abilities of PHLIP allow to automatically process large datasets. Although such processing may be computationally demanding, it is executed without the need of user intervention.

#### Statistical significance of results

Biofilm development is a stochastic process and, as a consequence, replicate experimental runs never produce the same biofilm structures. This leaves the issue of reproducibility usually open to the morphological quantification of biofilms from CLSM imaging [21]. The automation capabilities mentioned and the data structures used in the program architecture of PHLIP are specially suited to analyze large datasets with replicates, which is indispensable to obtain statistically significance from an experiment. The analysis of a marine phototrophic biofilm described here used this feature to provide a statistical evaluation of the experimental results.

#### Standardization

The presented XML format PHLIP-ML allows the program to work with CLSM data generated by any kind of microscope type. The portability and extensibility of XML offers many advantages to describe the complex nature of biological data and constitutes an important step towards the standardization of methods in biology [58-60]. The PHLIP-ML data structure was designed with the specific intent to serve as a scaffold for further extension to a CLSM standard for sharing data between image analysis programs or image databases. So far, the PHLIP-ML format only describes the microscopic parameters (pixel size, scanning step etc.), provides information about the CLSM data (number of samples/stacks) and stores calculated PHLIP image analysis results. The confocal images themselves only have their filenames identified in the PHLIP-ML data structure. In the future, a more general and self-contained approach for a CLSM standard format including a description of experimental procedures should be pursued. Due to the native extensibility of XML, it will be easy to upgrade the PHLIP-ML structure to meet the requirements of such a standard. This will provide a common description of the entire working flow ranging from image analysis to image acquisition process and represent a practical way to transparently reference imaging data to the biofilm community (publications, presentations etc).

#### Extensibility

The PHLIP package was built to be easily expandable by additional image processing functions and compatible to new input/output formats. Therefore, the open source project PHILP (made available at [34]) intends to serve as a framework for developers wishing to extend the program and implement their own image processing routines.

## Conclusion

PHLIP analysis was used in this study to reveal the dynamics of three components of a phototrophic biofilm: bacteria, micro-algae and EPS. The observed small contribution of bacteria in mature biofilms compared to algae and EPS suggests a shift from a heterotrophic to an autotrophic system during the biofilm development. Roughness of EPS was one of the morphological parameters measured. Its high value indicated the importance of EPS for the structure of the biofilm.

Enabling the quantification of biofilm morphogenesis as it is described here was the motivation to develop PHLIP. Throughout the analysis process of image segmentation, morphology description and data storage, PHLIP was designed to meet the requirements of single- and multi-channel capabilities of modern confocal laser scanning microscopy equipment. The PHLIP application software is made public available [53] as an open source implementation in the popular MATLAB scientific environment without the requirement of additional toolboxes. Its modular configuration was specifically developed to facilitate further extension with additional image processing functions in response to particular data analysis requirements and to support other data formats from various confocal microscope models. As examples of the application of image analysis techniques for the quantification of biofilm morphology become more common in the literature, the open structure of PHLIP intends to facilitate the development of novel image analysis procedures.

## Availability and requirements

PHLIP is distributed under an open source license and can be freely downloaded from the PHLIP project webpage [34]. The software comes as a MATLAB package (no toolboxes required) and runs on Linux, OSX and Windows platforms (see also implementation section).

## Authors' contributions

LM and JX programmed the presented image analysis software PHLIP. JB performed the experimental work and quantified the acquired imaging data using PHLIP. LM and JB wrote the manuscript. JA and LS participated in the design and coordination of the project and helped to draft this communication. All authors contributed to the presented study and approved the manuscript.

## Figures and Tables

**Table 1:** Minimum and maximum values for the image analysis parameters calculated by PHLIP during the development of phototrophic marine biofilms. ANOVA testing determined whether variations in biofilm parameters over time was statistically significant. Significance levels are indicated by asterisks. Channels; concanavaline A (ConA), syto64 (syto), chlorophyll*a *(chla).

	Single channel						Two channels						All channels	
			
	ConA		syto		Chla		ConA-syto		ConA-chla		syto-chla			
Biovolume^*a*^	5.8–28.8	n.s.	0.5–4.7	***	0.1–61.9	***							8.4–82.3	***
Substratum coverage	0.48–0.86	n.s.	0.07–1.63	***	0.03–2.86	***								
Area-to-volume ratio	3.5–5.8	n.s.	3.2–5.0	**	1.4–4.4	**								
Horizontal spreading^*a*^	8.4–9.1	n.s.	5.8–8.3	**	1.9–7.7	***								
Vertical spreading^*a*^	0.09–27.5	***	0.03–24.0	**	0.02–30.4	***								
Total spreading^*a*^	8.5–11.7	n.s.	8.3–9.3	*	2.0–10.8	***								
Average thickness	15.3–127.4	***	6.3–111.2	*	4.3–102.3	***							6.3–111.2	**
Biofilm roughness	0.15–0.26	n.s.	0.04–0.20	***	0.02–0.30	***							0.24–0.35	n.s.
Co-localization in 2D							5.4–14.5	n.s.	0–3.7	n.s.	0.5–27.9	n.s.		
Co-localization in 3D							0.9–5.2	n.s.	0–0.8	n.s.	0.04–2.7	n.s.		

*a*: × 10^3^; ***; p < 0.001; **: p < 0.01; *: p < 0.05; n.s. not significant

**Table 2:** Summary of the excitation wavelengths and emission filters for the multichannel signal of the CLSM using band pass (BP) and long pass (LP) filter.

Staining	Excitation [nm]	Emission filter [nm]
concanavaline A	488	580 ± 30 (BP)
syto64	568	600 (BP)
chlorophyll *a *(autofluorescence)	647	665 (LP)

**Table 3:** Feature overview of different image analysis software. Comparison of 2 recent published image analysis programs COMSTAT and ISA-3D with PHLIP. Feature information of COMSTAT [24, 26] and ISA-3D [38] was gathered from their reference publications (* data not published, ** data not available).

	**COMSTAT**	**ISA-3D**	**PHLIP**
Operating systems	Linux, Windows	Windows 2000/XP*	Linux, OSX, Windows
Requirements:	Matlab license + toolboxes	Matlab license + toolboxes	Matlab license only
Reference	[24, 26]	[54]	
Availability	Obtained free upon request	Must attend workshop (1500$)	Free downloadable (open source)
Multi-channel capability	No	No	Yes
GUI	No	Yes*	Yes
CLSM support	- Leica -COMSTAT	**	- Leica - COMSTAT - PHLIP-ML (universal converter)
Preprocessing	None	- Image inversion	- Image inversion - Carrier adjustment - X- section resolution
Automatic thresholding	Yes	Yes	Yes (Otsu)
Noise reduction:	Connected volume filtration	**	Connected volume filtration
Image analysis parameters:	- Single channel (10)	-Single channel (20)	- Single channel (6) - Two channel (2) - All channel (5)
Result output	- Text format	- Text format	- HTML format - XML format (PHLIP-ML) - Text-format
Source code availability	Not available	Not available	Available (open source project)
